# Professional autonomy and surveillance: the case of public reporting in cardiac surgery

**DOI:** 10.1111/1467-9566.12883

**Published:** 2019-03-15

**Authors:** Mark Exworthy, Jonathan Gabe, Ian R. Jones, Glenn Smith

**Affiliations:** ^1^ Health Services Management Centre University of Birmingham Birmingham UK; ^2^ Centre for Criminology and Sociology Royal Holloway University of London London UK; ^3^ School of Social Sciences Cardiff University Cardiff UK; ^4^ Imperial College London London UK

**Keywords:** public reporting, surveillance, autonomy, surgeons, medical profession

## Abstract

Professional autonomy has come under greater scrutiny due to managerialism, consumerism, information and communication technologies (ICT), and the changing composition of professions themselves. This scrutiny is often portrayed as a tension between professional and managerial logics. Recently, medical autonomy has increasingly been shaped in terms of transparency, where publication of clinical performance (via ICT) might be a more pervasive form of surveillance. Such transparency may have the potential for a more explicit managerial logic but is contested by clinicians. This paper applies notions of surveillance to public reporting of cardiac surgery, involving the online publication of mortality rates of named surgeons. It draws on qualitative data from a case‐study of cardiac surgeons in one hospital, incorporating interviews with health care managers and national policymakers in England. We examine how managerial logics are mediated by professional autonomy, generating patterns of enrolment, resistance and reactivity to public reporting. The managerial ‘gaze' of public reporting is becoming widespread but the surgical specialty is accommodating it, leading to a re‐assertion of knowledge, based on professional definitions. The paper assesses whether this form of surveillance is challenging to or being assimilated by the medical profession, thereby recasting the profession itself.

The autonomy of the medical profession (individually and collectively) has long been under scrutiny. It has come under further examination in the face of managerialism, consumerism, ICT (including the Internet) as well as the changing composition of the medical profession itself. These changes have generated substantial academic inquiry. In some cases, medical autonomy has been examined in isolation (Thomas and Hewitt 2011) but recent developments have meant that these issues have effectively coalesced under the broad title of transparency (Levay and Waks 2009). Although transparency seeks ‘to open practices to public scrutiny' (McGivern and Fischer 2012: 289), it can introduce surveillance as a loss of autonomy and a form of control of the (medical) profession (Exworthy 2015). However, it also introduces the possibility that the medical profession might accommodate it and (in some cases) enhance its legitimacy and status by it. How doctors react, respond and adapt is, therefore, crucial to the impact of transparency upon the profession, health services and patients.

This paper investigates the changing relationship between the medical profession and managers in England, and the strategies used by the latter to manage the former. We elaborate theoretical perspectives on professional and managerial logics in relation to the notions of autonomy, surveillance, soft bureaucracy and then evaluate them against data from a qualitative case‐study of public reporting of cardiac surgeons.

The paper is divided into three sections. First, using surveillance and medical practice to frame our analysis, we consider the dynamic relations between professional (primarily medical) autonomy and managerial logics, transparency and public reporting and forms of soft surveillance. Second, we report findings from an empirical study of public reporting in cardiac surgery in one English hospital and related stakeholders to explore this dynamic relationship. Finally, we draw conclusions about the wider reconfiguration of professional autonomy and surveillance.

## Medical autonomy and surveillance

The challenge to medical autonomy comes, in part, from a managerial logic. Here, this is examined in terms of new forms of surveillance in assessing medical performance. We explore how these developments find expression in cardiac surgery in England.

### Medical autonomy and a managerial logic

Recent studies of the articulation between professional autonomy and managerialism have focused on knowledge management, new forms of expertise, and evidence and performance (Numerato *et al*. 2012, Waring and Currie 2009). Much of this recent attention has focused on the tension between managerial and professional logics, denoting the underpinning set of ideas and values which produce and reproduce institutions (Martin *et al*. 2015). It is significant (for the analysis here) that logics can be applied at multiple levels, evolve over time and compete/interact with each other. As a result, logics can mutate and become hybrid. Arguably, in recent years, the professional logic has been in retreat in the face of managerial (and also market) logics. Yet, the latter's apparent ascendancy has prompted responses by the (medical) profession which point towards re‐professionalisation (including autonomy), albeit an uneven one which segments the profession more explicitly than perhaps ever before (Freidson 1994). Professionals thus undertake institutional work to challenge, maintain or reform these logics (Currie *et al*. 2012). Professions actively defend their autonomy, often through the creation, maintenance and transformation of institutions within which they work (Currie *et al*. 2012, Fournier 1999, Noordegraaf 2011). This may result in (re‐)professionalisation (emanating ‘from within' the profession) which can deliver “substantial” benefits to the profession (Elston 2009)

Doctors' reactions to managerial logics do, for example, alter the nature of medical expertise (Battilana 2011, Flynn 2002) and may facilitate the entry of a wider range of actors into the ‘performance' debate, which in turn, re‐adjusts professional hierarchies (Timmermans and Berg 1997). As medicine ‘colonises' new areas of expertise (*e.g*. performance measurement), it creates and enforces new social categories (*e.g*. the normative need to disclose work performance) (Suddaby and Viale 2011). Furthermore, digitisation is turning ‘knowledge into information that can be readily accessed' (Lupton 2015:21) and ICT can thus facilitate a ‘spectacle' or ‘gaze' whereby transparency of performance can be legitimated (Edelman 1988).

### Transparency and public reporting

The impetus towards greater transparency in health care (as part of a managerial logic) has emerged from the notion that medical professionals are not simply members of altruistic occupations but are also self‐serving interest groups (Freidson 1994, LeGrand 1997). In the UK, transparency in health care has been accelerated by the ‘naming and shaming' of ‘poor' performers, the Freedom of Information Act (2000; which has been applied in the UK since 2005), media reporting of medical scandals (*e.g*. Bristol Royal Infirmary and Mid‐Staffordshire hospital), patient consumerism, and choice/competition policies (Dixon‐Woods *et al*. 2011, Exworthy *et al*. 2010, Francis 2013, Hood and Heald 2006, Mannion and Davies 2002, McGivern and Fischer 2010, Pawson 2002). Transparency has thus become a ‘cornerstone of neo‐liberal accountability' (Koyama and Kania 2014) in England and many other countries (Blomgren 2007, Clarke and Oakley 2007, Gabe *et al*. 2012, Levay and Waks 2009).

Specifically, technological developments have enabled the collection of comparative information about professionals' performance, often previously resisted by doctors (Ackroyd *et al*. 1989, Exworthy 1998). By accumulating and making available comparable performance data on the Internet (Waring and Currie 2009), professional practice becomes auditable through commensuration and comparability (Espeland and Sauder 2007). However, the Internet‐based ‘picture' of performance may distort as much as reveal because the presentation, volume and complexity of data may hamper understandings of performance by some audiences (McGivern and Fischer 2012, Smith 1995, Tsoukas 1997). For example, textual information (usually accompanied by a graphical representation of data) often remains esoteric, using technical language and introducing caveats (such as risk‐based adjustments). This ambiguity is consistent with Suddaby and Viale's (2011) argument that professions disseminate new standards of performance and behaviour, which appear to be in the public interest but in formats that tend only to be understood by professionals themselves. Transparency can, nonetheless, reveal what has hitherto been neglected (or kept hidden) including poor or criminal practices.

As much routine clinical performance is hidden from external gaze (*e.g*. in the operating theatre), public reporting enables external agents to glean a picture of doctors' practice in order to control it. These ‘softwares' rely less on bureaucratic techniques of direct control and more on transforming agents (doctors) as ‘calculable selves' (Miller 1992) and an extension of the ‘gaze' of panoptic control (Exworthy 2015, Reed 1999). Although this might represent a shift (back) towards internalised disciplines of performance (*cf*. external control), it may also denote a growing prevalence of ‘micro level circuits of surveillance' (p.40). This carries a danger of overlooking systemic structures of power wrought by managerialism and other agendas (Kitchener *et al*. 2005).

### Soft surveillance and the spectacle of transparency

Theoretical models of professional control do not always easily accommodate the intersection between professional and managerial logics (Kitchener and Exworthy 2008, Martin *et al*. 2015). Here, we offer the notion of ‘soft surveillance', drawing on Courpasson's (2000) soft bureaucracy. The articulation between competing logics needs to reflect contextual nuances and how new forms of surveillance create a ‘loose coupling' between medical autonomy and managerial authority. Soft bureaucracy refers to the:

processes of flexibility and decentralisation [which] co‐exist with more rigid constraints and structures of domination (Courpasson 2000:57).

It entails an ambivalent structure with a ‘rigid exterior appearance and a loosely coupled set of interior practices' (Sheaff *et al*. 2003: 409). Professionals thus become involved in auditing their own practice (here, public reporting) but crucially do not suffer any (significant) loss of autonomy (*e.g*. performance management). When performance is made transparent, doctors often use three mechanisms to ‘define and defend' their performance: denial of actual mistakes, discounting by externalising the blame and distancing themselves from the event (Mizrahi 1984). If professionals' performance remains opaque or vague to external actors (despite the apparent move to transparency) (Courpasson 2000, Suddaby and Viale 2011), then their autonomy is not only preserved but possibly enhanced.

McGivern and Fischer (2010) examine the ‘spectacular transparency' of high profile and egregious cases of (medical) failure and subsequent regulation. Such transparency (involving the need to be seen to take action) contrasts with the ‘spectacle of transparency', which emphasises the need for demonstrable competence by professionals (Edelman 1988). Whilst this may help restore trust and preserve autonomy, it can also be a superficial form of compliance; a ‘tick box' approach against external interests, which engenders defensiveness (McGivern and Fischer 2012). Whether transparency internalises discipline or is a social defence mechanism, it has the capacity to re‐configure relations between the observed and the observer.

## Public reporting in cardiac surgery: a case‐study

Surgery is often seen as a prestigious speciality, given its ‘heroic' and ‘high‐tech', interventionist approaches (Fox 1992, Katz 1998). Cardiac surgery has pioneered Internet‐based public reporting in the UK (and elsewhere) (Chassin 2002, Keogh *et al*. 2004). Since 1994, cardiac surgery in England has published its annual audit database, originally in a paper format but since the early 2000s, on the Internet, with some hospitals initially acting as pilot sites (including the one in this paper). Yet, public reporting might prompt doctors to avoid high‐risk patients, fearing a negative impact upon their disclosed performance. Also, evidence suggests that patients do not generally use publicly reported clinical performance data to inform their decision‐making (Shekelle *et al*. 2008).

Public reporting has become a policy imperative in England over the last decade, becoming associated with policies fostering greater patient choice and competition, and advancing quality improvement (http://data.gov.uk/sites/default/files/Open_data_White_Paper.pdf; June 2012; http://www.data.gov.uk; http://theodi.org/drupal7/). Using ‘freedom of information' legislation, *The Guardian* newspaper published some clinical performance data in 2006. By 2007, the regulator, the Healthcare Commission (later re‐named Care Quality Commission), in conjunction with the Society of Cardiac‐Thoracic Surgeons (SCTS), published outcome results for individual surgical units in the National Health Service (NHS) in England (and some private hospitals). Since 2013, however, participation in public reporting across 10 specialties has been mandatory http://www.england.nhs.uk/2013/09/13/3special-consult-outcome-data/).

Public reporting of cardiac surgery has commonly measured the 30‐day post‐operative mortality rate. In the UK's 40 surgical centres, around 30,000 people have heart surgery each year (http://heartsurgery.cqc.org.uk/index.aspx), two‐thirds of whom have a coronary artery bypass graft (whose mortality rate was 1.05% in 2015; http://bluebook.scts.org/#PredictedRisk (isolated first‐time CABG)). Mortality measures apply to named, individual consultant cardiac surgeons who are held responsible for the clinical performance of junior surgeons for whom they are responsible. This neglects the whole clinical team's contribution. Cardiac surgery represents an ideal case for multi‐level, empirical research into the transparency of medical practice; the reactivity of professionals to such surveillance is set within organisational strategies and institutional ‘carriers' (Adler and Kwon 2013).

### Methods

We examined the enactment of public reporting by hospital surgeons (micro level), the mechanisms by which disclosed data were used by hospital managers and other actors (meso level), and the ways in which policymakers, regulators and surgical leaders managed the disclosure agenda (macro level) (Gabe *et al*. 2012, Griffiths 2003). These levels equate with Adler and Kwon's (2013) distinction between individual, organisation and institution, but are not wholly commensurate with a distinction between professional and managerial logics. This tiered, inter‐locking approach helps to explore the actions, reactions and interactions between actors as subjects and objects of surveillance at different levels and across competing logics. We conducted case‐study fieldwork in an English hospital between 2008 and 2010. At the *micro level,* five surgeons (three senior and two junior surgeons), one nurse and one data analyst (a clinician by background, who worked in the surgical department, collating surgeons' data) were interviewed. Interviews explored respondents' career paths, attitudes towards performance measurement and management, and the disclosure of data on the Internet. Each micro interview lasted about 75 minutes. Ten audit (‘morbidity and mortality' (M&M)) meetings, each lasting around 1 hour, were observed; these monthly meetings were held in private and discussed significant cases (usually death) amongst clinical colleagues. Three surgeons (who were also interviewed) were ‘shadowed' over 2 whole days; this involved observation in clinical (e.g. operating theatre) and administrative settings (e.g. team meetings) by one of the researchers. Both sets of observations enabled a greater understanding of the subjective experience of clinicians (especially relating to ways of managing performance), through everyday encounters between staff and a sensitisation to the surgical field. At the *meso level,* three interviews were conducted with hospital managers and those responsible for commissioning services from the hospital. These two groups reflected, respectively, internal and external sources of (apparent) control over surgeons. The former has been seen as a direct challenge to clinicians (Exworthy 2015, Harrison and Pollitt 1994) whereas the latter sought to effect change through market‐based mechanisms (Checkland *et al*. 2012). However, both internal and external sources have been limited in the impact (Kitchener and Exworthy 2008). Interviews explored the experiences of managing clinical performance, clinician‐management relations, trust in performance measures and the impact of published performance data upon decision‐making. These interviews lasted about 50 minutes. At the *macro level,* six interviews were conducted with representatives of professional bodies and regulators/policymakers. At this level, interviews explored the purpose of public reporting, the specialty's approach to managing clinical performance and regulatory regimes. Macro level interviews lasted about 60 minutes. In total, data collection comprised 16 interviews, observation of 10 M&M meetings, and shadowing for 3 days.

Ethical approval was secured from the NHS (London and Surrey Border research ethics committee; 08/H0806/47). Interviews were audio‐recorded (with permission) and transcribed in full. Observations were recorded by contemporaneous field‐notes. Transcripts and field‐notes were read independently by two researchers and a coding framework was devised which was then applied to all data. Qualitative software was not used given the size of the interview sample. Using a ‘framework' approach (Gale *et al*. 2013, Ritchie and Spencer 1994), we identified commonalities and differences in the data so as to explore inter‐connections between different levels, occupations and logics. We drew descriptive inferences based on *a priori* and emergent themes before elaborating conceptual perspectives. The empirical themes explored surgeons' interpretation of and responses to public reporting, as well as the meanings and motivations of other actors; these are presented below in terms of three sections: (i) surgeons' engagement with public reporting, (ii) surgeons' ownership of it and the potential for any loss of autonomy and (iii) the potential uses of published data by external actors as a way to control surgeons. Analytical triangulation was enhanced by feedback to the surgeons and to policymakers; both groups confirmed the data interpretation and conclusions. Pseudonyms are used below to preserve anonymity.

This empirical study is limited in that it draws inferences from data from one hospital. We did not interview patients (or their relatives/carers) and gathered limited data from non‐surgical clinical staff. However, data triangulation was achieved through interviews and observations over the course of a year. Furthermore, the tiered, multi‐level approach ensured that (senior and junior) surgeons' views at the micro level were nested within meso and macro levels; about half the interview sample was at the meso and macro levels, giving a wider perspective on the micro level. This nested approach helped to elaborate our theoretical perspective.

### Findings

#### Surgeons' enrolment in and resistance to public reporting

Explicit resistance was not commonly found at the micro level, possibly because the case‐study was one of the first English hospitals to introduce online surgical public reporting in the early 2000s; reporting had previously been paper‐based. However, micro level surgeons had a grudging acceptance of public reporting. Most (micro level) interviewees were critical of what one described as an ‘obsessive pursuit of auditing surgeons' (Oliver, data analyst). Subtle processes of resistance were, however, reported at the macro level, with senior professionals seeking to re‐shape the form of local public reporting. Passive resistance was expressed locally through the appointment of a data analyst by surgical department and the process of senior surgeons checking performance data before being passed to hospital managers. These actions seemed less to reject public reporting itself than to preserve (professional) control over its form and purpose. One senior surgeon (micro level) claimed that public reporting had no effect on his practice.

I have to say at my age I don't give a damn, I don't even look at my figures either. I've never visited any of these websites… and I will do what I think is best for the patient and if at some point my mortality is deemed to be unacceptable and then they put me out to grass…it has had no impact on me at all (Jack, surgeon).

At the macro level, surgeons noted the resistance from within the specialty to public reporting and other reforms but were more supportive of such reporting than micro level surgeons. For example, Brian offered a perspective across the specialty:

The most resistant people to that [i*.e*. public reporting] have been the cardiac surgeons themselves… at the minute, surgeons I think feel ‐ and other clinicians ‐ that this is being imposed on them (Brian, surgeon and policy‐maker).

Micro level resistance also consisted of refusing to disclose mortality rates as participation was (at that time) voluntary. Macro level resistance was much less apparent. According to two (macro level) respondents, about one‐sixth of all cardiac surgeons nationally did not disclose their mortality rates, given the voluntary nature of public reporting. They indicated that such non‐participation was concentrated in particular surgical departments (rather than evenly spread across the country). However, Bridgewater (2013), a surgeon, reassures that participation rates were ‘more than 96%', indicating either that surgeons were enrolled in public reporting and/or that they foresaw the compulsion to participate announced in 2013.

Damon suggests that the individual focus of reporting prompted resistance:

There are quite a few dissenting voices yeah, quite a few, the laggards… I suppose in some respects the individual [level of reporting] was a bridge too far, you know…. but it does up the ante quite a bit. If we set up with unit [*i.e*. the department level] report results, I suspect it would be a lot less heated (Damon).

The term ‘laggard' might be seen as pejorative (*i.e*. a quasi‐normative error; Bosk 2003) but Damon's qualification (favouring unit level reporting) suggests that such responses were understandable and acceptable.

Also, resistance took the form of resignations of (micro level) surgeons from their professional body.

This whole issue has been quite controversial and in some areas, some members have actually resigned from the Society (Luke).

These forms of resistance are significant because surgeons had largely designed and implemented the performance measure (Nashef 2015) and had designed the public reporting website with the regulator (see above). However, the examples of grudging acceptance amongst micro level surgeons may have reflected the timing of the fieldwork; that is, roughly 1 year into the pilot scheme at the hospital. Any initial resistance from surgeons might have evaporated, as Rupert implies.

There's also an aspect to which having the results published has been cathartic…any fall out has happened. We've done it over a number of years now and I think people are quite relaxed about it (Rupert, surgeon).

Micro level surgeons adopted rhetorical and instrumental tactics to justify their acceptance of and/or adaptation to public reporting. For example, some claimed that a trait of their surgical practice was self‐evaluation, which aligned with the aims of public reporting. They stressed the professional value of monitoring and audit:

Ask heart surgeons, we monitor ourselves very, very closely. We're very self‐critical, I think, of our own performance (Tom, junior surgeon).

Tom implied that such expertise had to be owned and led by surgeons, a point echoed by another junior surgeon:

I do very much agree with a point that performance needs to be monitored, because without monitoring of performance … then you don't really have an accurate idea as to where you're going, whether you're going through a difficult period, a bad patch (Ian, junior surgeon).

This was extended as far as an ethical dimension:

[Surgeons] have a duty – professional, moral and social responsibility ‐ to know what they do and how well they do it. (Brian).

Although not a transparency issue *per se*, this ‘responsibility' was used to justify professional ownership of public reporting and thereby a professional logic. The significance of ownership was recognised by a regulator who had worked with the SCTS in the development of public reporting:

Do you need somebody independent to develop it? Probably not because the clinicians themselves ‐ their involvement in the development of that is pretty important because actually you want it to be used [by surgeons] (Rob).

Such esoteric knowledge was also significant in surgeons' accounts of public reporting. One surgeon, for example, explained that his views about public reporting were contingent upon his level of experience:

I started as a consultant fortunately a couple of years before the figures became very public and very scrutinised. So I had a few years to build up a reputation… certainly if I'd started and if I knew that my first years' figures were going to be published with all the others, you know, in the annual report, it would have been very uncomfortable (Rupert).

Such comments imply a ‘protection' of junior surgeons from overt public scrutiny. Another (micro level) surgeon explained his socialisation into a ‘culture' of self‐evaluation and audit:

Everywhere I've been [working], there have been with people measuring what they did. It was initially very simple what they did; did they [patients] live or did they die? And so, that was the culture I grew up in. (Damon).

Micro level surgeons gave accounts which drew upon the emotional gravity of surgical operations as a justification for their specialty's ownership of public reporting.

I lost a patient recently and like most surgeons, you're sort of very upset and mortified by it …my colleagues were saying, ‘Look, it's just one of those things' and I was saying, ‘Well if this would have happened or that would have happened maybe this would have happened (Dave, junior surgeon).

In this case, ‘just one of those things' was a professional coping strategy when dealing with significant clinical incidents (Bosk 2003; Mizrahi 1984).

#### Surgeons' ownership of public reporting to avoid loss of autonomy

The move to national public reporting was opposed by some but it was made easier because surgeons' own scheme had become commonplace in the previous decade. Surgeons thus already ‘owned' public reporting and were well placed to ‘maintain' or ‘transform' it. In the context of recent medical scandals, this openness by surgeons was viewed as beneficial in restoring public trust:

I think it's [public reporting] protected the profession against criticisms of being, you know, secretive or, you know, their trying to hide behind, you know, patient confidentiality (Rupert).

Professional ownership of public reporting seemed to aid its uptake. Critical to this was voluntary participation. As one surgeon (Brian) explained, the regulator ‘never ever said that publication of individual surgeons' results should be mandatory.' Professional ownership equated with voluntary participation:

So we *have to have* some sort of measurement and far better that we do it and do it professionally and well, than have it imposed on us… I suppose some people would feel it's being imposed on us but I don't think that's… I think *we are still leading the way with it* and we get other people to help us with it… (Luke, emphasis added).

The balance between maintaining control of the initiative and securing surgeons' participation was widely appreciated because otherwise they feared a loss of autonomy:

The best approach is to say, leave it to us, stand aside and let us do it, we will do it properly, we will own it (Brian).

However, all surgeons felt that their own specialty's initiatives had been overtaken by policy developments, some of which they opposed. One surgeon indicated this ‘distancing' of surgeons from public reporting through a loss of control following the media's involvement:

I don't necessarily think it [being forced to publish data] is a particularly good idea, but we were boxed into it… we were into trying to get good measurements of results for individual units. What then happened was we were subject to a freedom of information act request from *The Guardian* [newspaper] (Brian).

Leaders of the surgical specialty claimed that public reporting helped to ‘defend' the profession. Having adopted disclosure, they argued, the need for explicit managerial initiatives was obviated.

The surgeons (especially senior surgeons at the micro level) recognised that autonomy was under threat from public reporting and regretted this change. They thus sought to retain this autonomy by emphasising the ownership of public reporting. This was manifest, for example, in terms of clinical responsibility of *individual* surgeons. David referred to an implicit ‘contract' of autonomy and responsibility:

Basically when I signed up to being a consultant, it was the buck would stop with me, you know. That was the deal in heart surgery, certainly… In some respects, many of us would not be keen that the buck would be stopping somewhere else because we have the autonomy to make a lot of decisions and things (David, surgeon).

The ‘somewhere else' implies performance management by external and/or managerial agents (Exworthy *et al*. 2003). Surgeons were fearful that public reporting of their ‘poor' results would harm their autonomy. For example, Damon feared publishing ‘negative results':

I'm sure it [public reporting] does make you vulnerable at having you reported to your Chief Executive or, you know, your Society [SCTS] if your figures take a blip… The fear of being suspended or fear of having a bad reputation is very real (Damon).

In observations, surgeons were open about their ‘negative' results but our field‐notes show tensions amongst clinicians in assessing surgical performance:

Members of the audience correct the presenter. One person suggests that the presentation is not what happened. …There doesn't seem to be an agreement. (Field‐notes, M&M meeting)

Most areas of dispute in the meetings we observed were ‘process' issues (*e.g*. the transfer of patients from another department) and were not related to specific procedures. However, one surgeon (Christopher) spoke, whilst being shadowed, of the ‘game playing' that took place in M&M meetings, highlighting the internal differences between and amongst staff groups. This reflects the assertion by Packwood *et al*. (1994); namely, senior clinicians (who tended to dominate discussions in M&M meetings) could ‘monitor' and ‘coach' junior doctors (who usually presented cases) about acceptable norms of behaviour. However, such behaviour was often implicit especially when the learning was not apparent but professional norms were being enacted by senior staff.

…the proceedings lack a palpable learning experience in which you could make sense of the discussion and could then apply it in practice. (Field‐notes, M&M meeting).

Such behaviour by seniors can thus reinforce existing professional practice rather than challenge it – the putative intention of M&M meetings and other ‘improvement' schemes. It can also shape the moral code of surgeons in terms of what constitutes an ‘acceptable' breach of it (Bosk 2003).

#### Potential uses of published data by external actors as a way to control surgeons

The English health policy narrative has emphasised the role of public reporting in enabling patients' choice of hospital (Fotaki 2006), integral to a market and managerial logic. However, when asked investigated how public reporting might help patients choose surgeons, (micro level) surgeons themselves were dismissive:

My experience is that very few patients tend to come and want to discuss the results or discuss the stuff on the website… and so I have to assume that's either because they find it interesting but irrelevant to their personal experience or they don't understand it… The fact that we put the data on the web is enough to satisfy the family (Rupert).

Micro level surgeons recognised that whilst few patients used the publicly reported data, it fostered patient trust (as Rupert and Damon indicated).

But they don't actually want to go and look at it, but they are reassured we are actually happy enough to publish it (Damon).

One commissioning manager (meso level) felt that the public did want to see published mortality data, and recognised that performance measurement was inevitable:

Patients will want to know mortality rates. Now whether that's separate from quality, that's fine. That's a debate to have but I think, you know, it's an important statistic to understand and it's always going to be measured (Peter, manager).

However, such an opinion was not supported by other (micro level) views. Some surgeons argued that the local commissioners of services ;didn't really ask us for results' (although they were available) but that the data had been used within the hospital. However, a hospital manager (meso level), Derek, acknowledged that the hospital has ‘done mortality [analysis] in isolation.' This implied that managers were content for surgeons to internalise this surveillance and were unwilling (or unable) to challenge surgeons as they (as a group) were starting to publish their results.

If you go on the internet, on our public website, we publish our mortality statistics and we're very proud of our performance… There have been a number of incidences when we have looked at the performance in recent times of our clinicians and investigated this (Derek, manager).

Derek might be implying a degree of (managerial) vigilance albeit involving a degree of delegation to the surgeons. His view contrasts with a senior surgeon who foresaw more extensive managerial control over the profession:

It's [public reporting] going to be built into the [individual] appraisal system… and it's going to be built into revalidation and that in turn will lead into service accreditation and then we are going to be looking at also ultimately ‐ leads into [hospital] trust board assurance (Brian).

Revalidation was indeed introduced in 2012 and involved the compulsory appraisal of individual doctor's performance as demonstrated in files and documents (Brennan *et al*., 2014). Although the relationship between revalidation and improved performance is uncertain (Greenhalgh and Wong, 2011), it does signal the institutionalisation of self‐governance. However, ‘complete self‐governance' may not always be complete, implying a partial hybrid logic (McGivern *et al*., 2015).

Arguably, the way in which (micro and macro level) surgeons used data was more significant as a mechanism to broadcast peer reputation (Hibbard *et al*. 2005).

First of all, mostly cardiac surgeons look at it, look at it extensively, and compare each other, so we look at it a lot; I suspect probably `health‐type' people look at it a bit. I suspect commissioners will look at it, but I suspect it won't change their practice… I suspect people like cardiologists and other surgeons probably look at it, but just out of interest (Damon).

Micro level surgeons were aware that operating on high‐risk patients might affect their publicly reported mortality rates. Yet invariably, they stated that they would operate nonetheless given the perceived problems with algorithms making risk predictions difficult. This helped to reinforce their professional autonomy.

Whilst being shadowed, all the surgeons spoke of the importance of teamwork in shaping their clinical performance in the operating theatre. In interviews, they claimed that mortality rates alone were poor markers of quality.

It [treating a high risk patient] would affect my published performance number, so yeah, so it would just make me unhappy. But it would not stop me doing it, and the reason I say it is because of the risk algorithms we have are relatively poor (Damon).

A key element to confound managerial logic of public reporting was to prevent ‘poor' performance from being reported in the first place. Thus, the spectacle of transparency was distorted because only ‘acceptable' performance was disclosed (Gabe *et al*. 2012). In this case‐study, this was partly enabled by the data analyst who liaised with senior surgeons about the data from the department.

Although surgical mortality rates had been declining for some time (SCTS, 2009), surgeons still retained significant leeway in defining performance measures and managing the consequences of it. One interviewee (micro level) spoke, for example, about a case of poor performance when the head of department spoke with the ‘errant' surgeon who was said to have changed his technique and apparently improved ‘his' performance (assuming a direct link between that surgeon's actions and patient mortality). Elsewhere, our observations revealed how senior surgeons shaped performance debates:

Jack [a senior surgeon] helps guide the questions and discussion and at times, tries to summarise that discussion in terms of what is learnt, but these good intentions are soon lost in the pressures of time (Field‐notes, M&M meeting).

The ‘quiet chat' and the ‘guidance' in M&M meetings signalled the various ways in which junior staff were socialised into normative behaviours of cardiac surgery (Bosk 2003, Rosenthal 1995). Although abusive behaviour was not observed in this study (*cf*. Bosk 2003), the power of senior surgeons in coaching juniors, and possibly obviating the putative effect of public reporting, was nonetheless apparent. Micro level surgeons minimised the impact of public reporting by referring to existing informal processes; peer reputation and pressure thus appeared stronger incentives.

If there was a potential problem with one of the surgeons, then some action would be taken to investigate it, and try and remedy any problems that were found *before* it got to such a stage where it was apparent to an outside observer that there was, you know, an obviously clear difference (Rupert; emphasis added).

Surgeons emphasised the socialisation process which excluded external scrutiny of processes which managed ‘poor' surgical performance. This esoteric knowledge negated the need, surgeons claimed, for external control or surveillance.

I think that takes a lot of wisdom to work out if somebody is having a bad year… I have a colleague at the minute who has pretty bad coronary results… and we have discussed the situation with him and told him he needs firstly to tighten up things for the next three months… Now in a month's time…, if the grass is growing the wrong way, there will be another discussion which will move from a discussion to this is what we need to do now (Damon).

We found no specific cases where surgeons deliberately changed their practice as a result of disclosure (although attribution would be hard to establish given our methodology). Avoidance of ‘high‐risk' patients would be a more subtle process which might involve a ;private conversation':

There is [sic] probably situations where I have a *private conversation* with individuals and they will say `I had two deaths in the past three months and I'm not going to take on anything risky for the next six months' (John; emphasis added).

## Discussion: autonomy and surveillance

The themes discussed above reveal the extent to which public reporting represents a significant shift in surgical professionalism whilst also opening new avenues of managerial control. Although (micro and macro levels) surgeons have largely maintained autonomy over performance monitoring, new forms of managerial scrutiny (including public reporting) have been developed. The putative intent of public reporting emphasised the transparency of surgical performance to other stakeholders; such a challenge to autonomy is redolent of a managerial logic. However, at the macro level, surgeons have influenced the adoption and spread of public reporting; at the meso level, they have shaped institutional procedures (such as clinical governance); and at the micro level, surgeons have continued to define professional customs and practices (*e.g*. through coaching junior surgeons)(Ackroyd *et al*. 1989). The ambivalence of some (micro level) surgeons to transparency belies the strategies of other (meso and macro level) surgeons who have mediated professional and managerial logics. Collectively, surgeons have managed to control the creation, maintenance and transformation of public reporting such that it offers less of a threat to their autonomy than an opportunity for the profession to enhance its position and status. This might suggest an ‘assimilation' of the managerial logic within the professional one, in which ‘core elements of the original logic prevail' (Martin *et al*. 2015:166). However, in some parts of the profession (mainly amongst leaders of the surgical profession), the managerial logic was being ‘blended' with the professional one. Furthermore investigation would be required to ascertain the subsequent balance of assimilation and blending across the surgical profession.

Stratification within the profession has, however, become more evident, with the rank‐and‐file surgeons becoming the subject and object of the transparent measures generated by the knowledge elite and enforced (normatively) by the administrative elite. This potentially threatens the cohesion of the profession if micro and macro levels become too detached, and if the managerial logic becomes too prominent. The notion of soft surveillance and ‘collaborative professionalism' (Adler *et al*. 2008) might mitigate this here since this study has shown that, despite outward signs of collaboration, the reactivity of surgeons transformed the policy intent of public reporting, deflecting the putative managerial logic in favour of a hybrid professional‐managerial one. Adler and Kwon's (2013) notion of a mutating profession might thus be more appropriate than collaborative professionalism, as rank‐and‐file surgeons seemed to tolerate a loss of autonomy for some institutional control in the form of surveillance. Although new performance regimes have been created and hierarchies within the surgical specialty have been re‐defined, the reconfiguration of expertise and autonomy is significant here. Surgeons have largely been able to deflect an overt managerial logic through a re‐definition of their expertise. Although demonstrable competence has necessitated reporting of mortality rates, this has helped to ‘define a new, open and uncontested space' for surgeons (Suddaby and Viale 2011:423) which helps to maintain some autonomy, to re‐establish legitimacy of their rules and status, and to resist explicit managerial logics.

The discourse and ostensible practice of public reporting of surgery was initially coupled (albeit loosely) to managerial logics. Despite the rhetoric of patient choice and competition in English health policy (akin to a market logic), managers (and patients) appear to have neither wanted nor been able to exercise direct control of surgeons' performance through public reporting. Surgeons have acceded to some loss of autonomy through the accumulation and storage of performance data (one measure) by the regulator (jointly with their professional body). Public reporting thus offers the surgical profession a strategy to enhance the autonomy of some of its members, in part by assimilating and blending some managerial logics to create emergent hybrid logics.

Public reporting of surgery reveals a tension between the measurement of expertise (in terms of mortality rates) and the transformation of the profession's own jurisdiction. This is the essence of soft surveillance; the articulation between transparency and (professional) autonomy. If surveillance is implemented and ‘regulated' by the profession itself, surgical autonomy can be preserved or even enhanced. This confluence might also legitimise the specialty through the development of a new professionalism and an apparent restoration of public trust (Calnan and Rowe 2008, Elston 2009). However, in doing so, even soft surveillance requires greater engagement of surgeons with ‘reactivity mechanisms' (Espeland and Sauder 2007), including the normalisation of transparency. Such reflexivity may, however, create the potential for manipulation of data and/or surgeon behaviour, and defensive responses (Mizrahi 1984).

## Conclusion

Managing clinical performance continues to be interpreted and enacted through multiple means, One such means has been public reporting, located at the cusp of professional and managerial logics and with the potential for contestation between the two. An empirical illustration within a high profile specialty may therefore be expected to reveal the consequences of public reporting more generally. In this paper, we have argued that the strong professional boundaries of cardiac surgery combined with a relatively weak institutional framework are likely to inhibit the potential of public reporting as a form of transparency. The managerial logics have been assimilated and blended, thereby re‐shaping the surgical profession. Thus, we suggest, soft surveillance is evident whereby the surgical specialty has been able to create, maintain and transform public reporting according to its own professional logics of autonomy and expertise. That said, to secure its legitimacy in controlling the management of its own performance, the profession has blended with some managerial logics by engaging in the spectacle of transparency.

This case‐study of public reporting in cardiac surgery has shed light on the evolving nature of professionalism within large‐scale bureaucracies at a micro level of surgical practice, a meso level of organisational governance, and a macro institutional level as well as the reactivity between these levels and the nature of public reporting itself. It has also demonstrated the adaptability of expertise and practice at different levels of the surgical specialty in re‐defining managerial challenges of new softwares of control. By reacting to transparency and transforming an indicator of their expertise, surgeons have reconstructed their professional jurisdiction. Whilst transparency offers the potential for external control (and ‘control at a distance'), this has largely been ineffectual to date. The most significant impact has been the introduction of a new normative logic *within* the profession.

## References

[shil12883-bib-0001] Ackroyd, S. , Hughes, J. and Soothill, K. (1989) Public sector services and their management, Journal of Management Studies, 26, 6, 603–19.

[shil12883-bib-0002] Adler, P. and Kwon, S.W. (2013) The mutation of professionalism as a contested diffusion process: clinical guidelines as carriers of institutional change in medicine, Journal of Management Studies, 50, 5, 930–62.

[shil12883-bib-0003] Adler, P. , Kwon, S.W. and Heckscher, C. (2008) Professional work: the emergence of collaborative community, Organization Science, 19, 2, 359–76.

[shil12883-bib-0004] Battilana, J. (2011) The enabling role of social position in diverging from the institutional status quo: evidence from the UK National Health Service, Organization Science, 22, 4, 817–34.

[shil12883-bib-0005] Blomgren, M. (2007) The drive for transparency: organizational field transformations in Swedish health care, Public Administration, 85, 1, 67–82.

[shil12883-bib-0006] Bosk, C . (2003) Forgive and Remember: Managing Medical Failure. Chicago: University of Chicago Press. 2nd edition.

[shil12883-bib-0500] Brennan, N. , Bryce, M. , Pearson, M. , Wong, G. , *et al* (2014) Understanding how appraisal of doctors produces its effects: a realist review protocol, BMJ Open, 4, e005466. 10.1136/bmjopen-2014-005466 PMC4067866 24958211

[shil12883-bib-0007] Bridgewater, B . (2013) `Consultant‐level outcomes reporting: the facts about consent.' Available at http://hqip.org.uk/consultant-level-outcomes-reporting-the-facts-about-consent/ (Last accessed 13 June 2013).

[shil12883-bib-0008] Calnan, M. and Rowe, R. (2008) Trust Matters in Health Care. Maidenhead: Open University Press.

[shil12883-bib-0009] Chassin, M.R. (2002) Achieving and sustaining improved quality: lessons from New York State and cardiac surgery, Health Affairs, 21, 4, 40–51. 12117152 10.1377/hlthaff.21.4.40

[shil12883-bib-0010] Checkland, K. , Harrison, S. , Snow, S. , McDermott, I. , *et al* (2012) `Commissioning in the English National Health Service: what's the problem?, Journal of Social Policy, 41, 3, 533–50.

[shil12883-bib-0011] Clarke S. and Oakley J. (eds) (2007) Informed Consent and Clinician Accountability: The Ethics of Report Cards on Surgeon Performance. Cambridge: Cambridge University Press.

[shil12883-bib-0012] Courpasson, D. (2000) Managerial strategies of domination: power in soft bureaucracies, Organization Studies, 21, 1, 141–61.

[shil12883-bib-0013] Currie, G. , Lockett, A. , Finn, R. , Martin, G. , *et al* (2012) Institutional work to maintain professional power: recreating the model of medical professionalism, Organization Studies, 33, 7, 937–62.

[shil12883-bib-0014] Dixon‐Woods, M. , Yeung, K. and Bosk, C.L. (2011) Why is UK medicine no longer a self‐regulating profession? The role of scandals involving `bad apple' doctors, Social Science and Medicine, 73, 10, 1452–9. 21975027 10.1016/j.socscimed.2011.08.031

[shil12883-bib-0015] Edelman, M. (1988) Constructing the Political Spectacle. Chicago: University of Chicago Press.

[shil12883-bib-0016] Elston, M.‐A. (2009) Re‐making a trustworthy medical profession in twenty‐first century Britain In Gabe J. and Calnan M. (eds) The New Sociology of the Health Service. London: Routledge.

[shil12883-bib-0017] Espeland, W.N. and Sauder, M. (2007) Rankings and reactivity: how public measures recreate social worlds, American Journal of Sociology, 113, 1–40.

[shil12883-bib-0018] Exworthy, M. (1998) Clinical audit in the NHS internal market: from peer review to external monitoring, Public Policy and Administration, 13, 2, 40–53.

[shil12883-bib-0019] Exworthy, M . (2015) The iron cage and the gaze: interpreting medical control in the English health system. Professions and Professionalism, 5, 1. Available at https://journals.hioa.no/index.php/pp/article/view/944

[shil12883-bib-0021] Exworthy, M. , Wilkinson, E.K. , McColl, A. , Moore, M. , *et al* (2003) The role of performance indicators in changing the autonomy of the general practice profession in the UK, Social Science and Medicine, 56, 1493–4. 12614700 10.1016/s0277-9536(02)00151-x

[shil12883-bib-0022] Exworthy, M. , Smith, G. , Gabe, J. and Jones, I.R. (2010) `Open heart surgery? The whys and wherefores of disclosing clinical performance data', Journal of Health, Organization and Management, 24, 6, 571–83. 21155433 10.1108/14777261011088665

[shil12883-bib-0023] Flynn, R. (2002) Clinical governance and governmentality, Health, Risk and Society, 4, 2, 155–73.

[shil12883-bib-0024] Fotaki, M. (2006) Choice is yours: a psychodynamic exploration of health policymaking and its consequences for the English National Health Service, Human Relations, 59, 12, 1711–44.

[shil12883-bib-0025] Fournier, V. (1999) The appeal to ‘professionalism' as a disciplinary mechanism, Sociological Review, 47, 2, 280–307.

[shil12883-bib-0027] Fox, N. (1992) The Social Meaning of Surgery. Buckingham: Open University Press.

[shil12883-bib-0028] Francis, R . (chair) (2013) Report of the Mid Staffordshire NHS Foundation Trust Public Inquiry. Volume 1: Analysis of Evidence and Lessons Learnt. HC‐898‐1. London: The Stationery Office.

[shil12883-bib-0029] Freidson, E. (1994) Professionalism: The Third Logic. Chicago: University of Chicago Press.

[shil12883-bib-0030] Gabe, J. , Exworthy, M. , Jones, I.R. and Smith, G. (2012) Towards a Sociology of Disclosure: the case of surgical performance, Sociology Compass, 6, 11, 908–22.

[shil12883-bib-0031] Gale, N. , Heath, G. , Cameron, E. , Rashid, S. , *et al* (2013) `Using the Framework Method for the analysis of qualitative data in multi‐disciplinary health research', BMC Medical Research Methodology, 13, 117. 24047204 10.1186/1471-2288-13-117 PMC3848812

[shil12883-bib-0501] Greenhalgh, T. and Wong, G. (2011) Revalidation: a critical appraisal, British Journal of General Practice, 61, 584, 166–8. 21375899 10.3399/bjgp11X561113 PMC3047308

[shil12883-bib-0032] Griffiths, L. (2003) Making connections: studies of the social organisation of healthcare, Sociology of Health and Illness, 25, 155–71. 14498935 10.1111/1467-9566.00345

[shil12883-bib-0033] Harrison, S. and Pollitt, C. (1994) Controlling Health Professionals. Buckingham: Open University Press.

[shil12883-bib-0034] Hibbard, J. , Stockard, J. and Tusler, M . (2005) Hospital performance reports: impact on quality, market share, and reputation. Health Affairs, 24, 1150–60. 10.1377/hlthaff.24.4.1150 16012155

[shil12883-bib-0035] Hood C. and Heald D. (eds) (2006) Transparency; the Key to Better Governance. Oxford: British Academy/Oxford University Press.

[shil12883-bib-0036] Katz, P. (1998) The Scalpel's Edge: The Culture of Surgeons. Boston, MA: Allyn and Bacon.

[shil12883-bib-0037] Keogh, B. , Spiegelhalter, D. , Bailey, A. , Roxburgh, J., *et al* (2004) The legacy of Bristol: public disclosure of Individual surgeons' results, British Medical Journal, 329, 450–4. 15321906 10.1136/bmj.329.7463.450 PMC514215

[shil12883-bib-0038] Kitchener, M. and Exworthy, M. (2008) Models of medical work control: a theory elaboration from English general practice In McKee L., Ferlie E. and Hyde P. (eds) Organising and Re‐Organising: Power and Change in Health‐Care Organisations. Basingstoke: Palgrave Macmillan.

[shil12883-bib-0039] Kitchener, M. , Caronna, C.A. and Shortell, S.M. (2005) From the doctor's workshop to the iron cage? Evolving modes of physician control in US health systems, Social Science and Medicine, 60, 1311–22. 15626526 10.1016/j.socscimed.2004.07.008

[shil12883-bib-0040] Koyama, J.K. and Kania, B.K. (2014) When transparency obscures: the political spectacle of accountability, Journal for Critical Education Policy Studies, 12, 1, 143–69.

[shil12883-bib-0041] LeGrand, J. (1997) Knights, knaves or pawns? Human behaviour and social policy, Journal of Social Policy, 26, 2, 149–69.

[shil12883-bib-0042] Levay, C. and Waks, C. (2009) Professions and the pursuit of transparency in healthcare: two cases of soft autonomy, Organization Studies, 30, 5, 509–27.

[shil12883-bib-0044] Lupton, D. (2015) Digital Sociology. London: Routledge.

[shil12883-bib-0045] Mannion, R. and Davies, H.T.O. (2002) Reporting health care performance: learning from the past, prospects for the future, Journal of Evaluation in Clinical Practice, 8, 215–28. 12180369 10.1046/j.1365-2753.2002.00331.x

[shil12883-bib-0046] Martin, G.P. , Armstrong, N. , Aveling, E.‐L. , Herbert, G. , *et al* (2015) Professionalisation redundant, reshaped or reinvigorated? Realizing the `third logic' in contemporary health care, Journal of Health and Social Behaviour, 56, 3, 378–97. 10.1177/0022146515596353 PMC4826742 26276676

[shil12883-bib-0047] McGivern, G. and Fischer, M. (2010) Medical regulation, spectacular transparency and the blame business, Journal of Health Organization and Management, 24, 6, 597–610. 21155435 10.1108/14777261011088683

[shil12883-bib-0048] McGivern, G. and Fischer, M. (2012) Reactivity and reactions to regulatory transparency in medicine, psychotherapy and counselling, Social Science and Medicine, 74, 289–96. 22104085 10.1016/j.socscimed.2011.09.035

[shil12883-bib-0502] McGivern, G. , Currie, G. , Ferlie, E. , Fitzgerald, L. , *et al* (2015) 'Hybrid manager‐professionals' identity work: the maintenance and hybridization of medical professionalism in managerial contexts, Public Administration, 93, 2, 412–32.

[shil12883-bib-0049] Miller, P . (1992) Accounting and subjectivity: the invention of calculating selves and calculating spaces. Annals of Scholarship, 9, 1/2, 61–86.

[shil12883-bib-0050] Mizrahi, T. (1984) Managing medical mistakes: ideology, insularity and accountability among internists in training, Social Science and Medicine, 19, 2, 135–46. 6474229 10.1016/0277-9536(84)90280-6

[shil12883-bib-0051] Nashef, S. (2015) The Naked Surgeon: The Power and Perils of Transparency in Medicine. London: Scribe.

[shil12883-bib-0052] Noordegraaf, M. (2011) Risky business: how professionals and professional fields (must) deal with organizational issues, Organization Studies, 32, 10, 1349–71.

[shil12883-bib-0053] Numerato, D. , Salvatore, D. and Fattore, G. (2012) The impact of management on medical professionalism: a review, Sociology of Health and Illness, 34, 4, 626–44. 21929618 10.1111/j.1467-9566.2011.01393.x

[shil12883-bib-0054] Packwood, T. , Kerrison, S. and Buxton, M. (1994) The implementation of medical audit, Social Policy and Administration, 28, 4, 299–316.

[shil12883-bib-0055] Pawson, R . (2002) Evidence and policy and naming and shaming. Policy Studies, 23, 3/4, 211–30

[shil12883-bib-0056] Reed, M. (1999) From the cage to the gaze? The dynamics of organizational control in late modernity In Morgan G. and Engwall L. (eds) Regulations and Organizations: International Perspectives. London: Routledge.

[shil12883-bib-0057] Ritchie, J. and Spencer, L. (1994) Qualitative data analysis for applied policy research In Bryman A. and Burgess R.G. (eds) Analyzing Qualitative Data. London: Routledge.

[shil12883-bib-0058] Rosenthal, M. (1995) The Incompetent Doctor: Behind Closed Doors. Buckingham: Open University Press.

[shil12883-bib-0059] Sheaff, R. , Rogers, A. , Pickard, S. , Marshall, M. , *et al* (2003) A subtle governance: `soft' medical leadership in English primary care, Sociology of Health and Illness, 25, 5, 408–28. 14498918 10.1111/1467-9566.00352

[shil12883-bib-0060] Shekelle, P. , Lim, Y.‐W. , Mattke, S. and Damberg, C. (2008) Does Public Release of Performance Results Improve Quality of Care?. London: Health Foundation.

[shil12883-bib-0061] Smith, PC . (1995) On the unintended consequences of publishing performance data in the public sector. International Journal of Public Administration, 18, 2 and 3, 277–310.

[shil12883-bib-0062] Society of Cardiac‐Thoracic Surgeons (2009). Available at http://www.scts.org/sections/audit/Cardiac/index.html

[shil12883-bib-0063] Suddaby, R. and Viale, T. (2011) Professions and field‐level change: institutional work and the professional project, Current Sociology, 59, 4, 423–42.

[shil12883-bib-0064] Thomas, P. and Hewitt, J. (2011) Managerial organization and professional autonomy: a discourse‐based conceptualization, Organization Studies, 32, 10, 1373–93.

[shil12883-bib-0065] Timmermans, S. and Berg, M. (1997) Standardization in action: achieving local universality through medical protocols, Social Studies of Science, 27, 2, 273–305.

[shil12883-bib-0066] Tsoukas, H. (1997) The tyranny of light, Futures, 29, 9, 827–43.

[shil12883-bib-0067] Waring, J. and Currie, G. (2009) Managing expert knowledge: organizational challenges and managerial futures for the UK medical profession, Organization Studies, 30, 7, 755–78.

